# Clinical values of expression signature of circCDR1AS and circHIAT1 in prostate cancer: Two circRNAs with regulatory function in androgen receptor (AR) and PI3K/AKT signaling pathways

**DOI:** 10.1002/jcla.24220

**Published:** 2022-01-10

**Authors:** Mahsa Aghajani Mir, Hossein Dinmohammadi, Emadoddin Moudi, Nima Motamed, Abdolreza Daraei

**Affiliations:** ^1^ Department of Genetics and Molecular Medicine Faculty of Medicine Zanjan University of Medical Sciences Zanjan Iran; ^2^ Department of Genetics and Molecular Medicine School of Medicine Zanjan University of Medical Sciences Zanjan Iran; ^3^ Department of Urology Babol University of Medical Sciences Babol Iran; ^4^ The Faculty Member of the Department of Social Medical Social Determinants of Health Research Center Zanjan University of Medical Sciences Zanjan Iran; ^5^ Cellular and Molecular Biology Research Center Health Research Institute Babol University of Medical Sciences Babol Iran

**Keywords:** circCDR1AS, circHIAT1, expression, prostate Cancer

## Abstract

**Background:**

Prostate cancer (PCa) is a genetically heterogeneous disease with highly molecular aberrations. It has been revealed that a newly discovered class of non‐coding RNAs called circular RNAs (circRNAs) play key roles in dictating tumor behaviors and phenotypes of the prostate tumors. In the current study, our aim was to determine the expression profiles of circHIAT1 and circCDR1AS in PCa compared with benign prostatic hyperplasia (BPH) tissues, as well as their clinicopathological relevance.

**Methods:**

The 50 prostate tissues including 25 PCa tissues and 25 BPH samples were collected for analyzing the expression levels of target circRNAs by quantitative real‐time PCR (qRT‐PCR).

**Results:**

The results revealed that expression of circCDR1AS was significantly elevated in PCa compared with the BPH (*p* < 0.05). We also observed that PCa patients over the age of 60 had a higher expression of the circCDR1AS than patients under the age of 60 (*p *= 0.017). Moreover, a lower expression level of circHIAT1 was found in the PCa than BPH tissues (*p* < 0.05), and finally, the findings indicated that the area under the curve (AUC) of circCDR1AS was 0.848, with 92% sensitivity and 76% specificity, as well as an AUC of 0.828, with the 80% sensitivity and 76% specificity for circHIAT1.

**Conclusion:**

These observations suggest that the abnormal expression of circCDR1AS and circHIAT1 can be regarded as two different types of molecular pathology with potential biomarker values for PCa, although further studies are needed.

## Graphical abstract

The AR and PI3K/AKT are the most commonly deregulated pathways in PCa. Interestingly, there is evidence that two key circRNAs, circHIAT1 and circCDR1AS, play important roles in tumor development by controlling AR and PI3K/AKT signaling pathways, respectively. In current study, the expression of circCDR1AS was significantly elevated in Prostate cancer (PCa) compared with the benign prostatic hyperplasia (BPH) (*p* < 0.05). We also observed a lower expression level of circHIAT1 was found in the PCa than in BPH tissues (*p* < 0.05). Moreover, potential diagnostic biomarker values analysis detected an area under the curve (AUC) of 0.848, with 92% sensitivity and 76% specificity for circCDR1AS and an AUC of 0.828, with the 80% sensitivity and 76% specificity for circHIAT1.

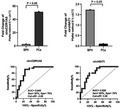

## 1 INTRODUCTION

Prostate cancer (PCa) is of great clinical importance in terms of both its prevalence and mortality in men globally.^1^ Despite the significant advances that have been made in the diagnosis and treatment of this cancer, it still has heavy costs on the health systems of different countries in the world.^2^ The phases of formation, development, and progression of PCa are driven by heterogeneous molecular changes that phenotypically dictate the heterogeneous behaviors and pathophysiological features of prostate tumors.^3^ On the contrary, there is limited evidence for the early and accurate detection of PCa.^4^ Therefore, it seems that the best goal to find new and more specific and sensitive diagnostic, therapeutic and prognostic biomarkers is to better understand the molecular pathophysiology of this cancer by revealing tumor‐specific genetic changes and linking them to emerging phenotypes.^3^, ^5^

Thanks to advanced expression analysis technologies, it has been revealed that one of the key such molecular changes that play important roles in the pathophysiology of cancers is the abnormal expression of a newly discovered class of non‐coding RNA (ncRNAs) called circular RNAs (circRNAs); however, little is known about their roles in PCa.^6^, ^7^ In this regard, there are some clues that abnormalities in the circRNAs can manifest themselves through dysregulating the important signaling pathways involved in the development of tumorigenesis, such as androgen receptor (AR) and PI3K/AKT pathways.^8^, ^9^ The AR and PI3K/AKT are the most commonly deregulated pathways in PCa.^10^, ^11^, ^12^ The abnormal implication of the AR signaling in PCa is executed via modulating the cell proliferation, survival, and invasion in early and late tumors.^12^ In addition, intensification and activation of signaling from the PI3K/AKT pathway in PCa have been shown to lead to resistant, metastatic, and aggressive phenotypes in prostate tumors by regulating the metabolism, survival, growth, progression, and cytoskeleton reorganization of tumor cells.^13^ There is also evidence of reciprocal regulatory interaction between members of these two signaling pathways, which has been shown to play an important role in PCa development and progression.^12^ Accordingly, the key to these two pathways in PCa has led to the development of some potent AR and PI3K/AKT targeted drug treatments.^12^

Interestingly, there is evidence that two key circRNAs, circHIAT1 and circCDR1AS, play important roles in tumor development by controlling AR and PI3K/AKT signaling pathways, respectively.^14^, ^15^ For example, published data have shown that circCDR1AS is overexpressed in gastric cancer and hepatocellular carcinoma (HCC) and through regulating some target genes in the PI3K/AKT pathway, promotes cell proliferation and invasion of the tumor cells.^15^, ^16^ In the case of PCa, only data on the circCDR1AS are available, and a new study on the PCa cell lines has revealed that this circRNA plays a prominent role in the invasion and migration tumor cells by suppressing miR‐641.^17^ Structurally, circCDR1AS is a highly conserved single‐chain circular RNA with length of 1485 nucleotide and without 5’ cap and 3’ poly‐A tail structure.^18^, ^19^ CDR1as shows tissue‐time specific expression, and because of its regulatory strong effects on the miR‐7, it is co‐expressed with this miRNA in the brain.^19^, ^20^ Under physiological conditions, it has the highest expression levels in brain tissue and spinal cord, and the lowest in the lungs, muscles, and heart.^18^ Pathologically, changes in its expression also occur in some diseases, including the pulmonary fibrosis, myocardial infarction, and cancers mentioned above.^21^, ^22^, ^23^ The circHIAT1 has a spliced sequence length of 807 nucleotides that is transcribed from its host gene called hippocampus abundant transcript 1 (HIAT1). The HIAT1 gene encodes a transmembrane protein of unknown function with homology to the solute carrier protein family and may transport a solute required for spermatogenesis from the bloodstream.^24^ A previous study has reported that expression of circHIAT1 is downregulated by AR in cancer cell lines and tumor tissue of clear cell renal cell carcinoma (ccRCC) cancer, and this led to increased proliferation, migration, and invasion of tumor cells in vitro and in vivo, as well as poor clinical features, including a worse overall survival of the patients.^14^ Therefore, given the important involvement of AR and PI3K/AKT pathways in the development of prostate tumors and also the existence of such key clues about the regulatory roles of circHIAT1 and circCDR1AS in these pathways, we hypothesized that the expression levels of these two circRNAs could be altered in prostate tumors in relation to patients’ clinical phenotypes. Accordingly, this study was aimed to determine the expression profiles of circHIAT1 (hsa_circ_0000096) and circCDR1AS (hsa_circ_0001946) in prostate tumor tissues as well as their correlations with clinicopathological features of the patients.

## 2 MATERIALS AND METHODS

### 2.1 Tissue samples

In this research, a set of 50 prostate puncture tissues including 25 prostate tumor tissues and 25 benign prostatic hyperplasia (BPH) tissues were taken from the subjects by transrectal biopsy procedure in Shahid Beheshti hospital and Babol Clinic Hospital Affiliated to the Babol University of Medical Science between April 2020 and November 2021. Furthermore, none of the patients underwent any treatment, including chemotherapy, radiotherapy, and targeted therapy before transrectal biopsy procedure. The selection of PCa and BPH subjects and the preparation of samples from them was based on clinical criteria for differential diagnosis of PCa and BPH by an urologist. Each tissue was confirmed by a PCa pathologist according to Gleason scores. Inclusion criteria for biopsy were a PSA level higher than normal based on age. The clinical information of all samples was collected, and the PCa and BPH diagnosis was finally confirmed by histopathology. The histological grades were assessed according to the American Joint Committee on Cancer (AJCC) cancer staging manual (8th edition).^25^ After sampling, each sample was divided into two parts, one for pathological examination and diagnosis of PCa and BPH and the other was immediately snap frozen in the liquid nitrogen (−196 C°) in nitrogen and sent to the molecular laboratory and then stored at −80 C° until RNA extraction phase. Of note, a written informed consent was gained from each study participant. This research was approved by the Ethics Committee of the Zanjan University of Medical Sciences.

### 2.2 Data collection

The clinicopathological characteristics, age, BMI, DRE, PSA level before surgery, Free/total PSA ratio, PSA density, Gleason score, ISUP, bone metastasis, and Family history of PCa obtained from medical reports (Table 1).

**TABLE 1:** Baseline characteristics of the study participants

Characteristics	Subgroup	Number (%)		*p* value
		BPH	PCa	
Age (years)	≤60	4 (16)	5 (20)	0.364
	>60	21 (84)	20 (80)	
Body mass index (BMI) (kg/m²)	18.5–24.9	6 (24)	11 (44)	0.142
	25–29.9	11 (44)	9 (36)	
	≥30	8 (32)	5 (20)	
Digital rectal examination (DRE)	Normal	21 (84)	11 (44)	**0.004**
	Abnormal	4 (16)	14 (56)	
Total prostate‐specific antigen (PSA) (ng/ml)	<4	6 (28.57)	1 (4.35)	**<0.001**
	4–9.9	10 (47.62)	5 (21.73)	
	10–20	3 (14.29)	6 (26.09)	
	>20	2 (9.52)	11 (47.83)	
Free/total PSA ratio (ng/ml %)	≤10	3 (30)	6 (42.86)	0.112
	11–18	2 (20)	7 (50)	
	18.1–25	2 (20)	1 (7.14)	
	>25	3 (30)	0	
PSA density (PSAD) (ng/ml/cm^3^)	≤0.15	18 (81.82)	9 (39.13)	**0.004**
	>0.15	4 (18.18)	14 (60.87)	
Gleason score (GS) (*n*/%)	≤6	‐	4 (16)	‐
	7	‐	12 (48)	
	≥8	‐	9 (36)	
International society of urological pathology (ISUP) (*n*/%)	1	‐	4 (16)	‐
	2	‐	1 (4)	
	3	‐	11 (44)	
	4	‐	9 (36)	
Bone metastasis	Negative	‐	14 (73.68)	0.375
	Positive	‐	5 (22.26.32)	
Family history of PCa	Yes	21 (84)	11 (44)	0.050
	No	4 (16)	14 (56)	

Note

Data were presented as count or percentage. PCa; prostate Cancer. BPH; benign prostatic hyperplasia. *p* < 0.05 was considered significant (in bold). For some variables, including prostate‐specific antigen (PSA) levels prior to surgery, free/total PSA ratio, PSA density, and bone scan, the data in the patients’ medical records were incomplete.

### 2.3 Total RNA extraction and complementary DNA (cDNA) synthesis

Total RNA was prepared from prostate tissues using SamBio kit (Cat NO. Sam004, South Korea) according to the manufacturer's protocol. The RNA concentration of each sample was measured with Nanodrop 2000 (Thermo Scientific, MA, USA). Total RNA integrity and gDNA contamination were measured by denaturing agarose gel 1.2% electrophoresis. cDNA was synthesized from 500 ng total RNA with the BioFACT Kit (cat NO. BR631‐050, German) according to the manufacturer's protocol.

### 2.4 Quantitative real‐time polymerase chain reaction (RT‐qPCR)

The real‐time PCR analyses of the expression level of the circRNAs were performed by using SYBR Green BioFACT 2x Master Mix (high ROX, German). qRT‐PCR for circRNA was performed on QIAGEN Rotor‐Gene Q real‐time PCR and the related specific primers were as follows: circCDR1AS, forward: CCAGAAAGTGTTGCAGCGTT, reverse: CCAAGGTGGGTGCTGTCAAT; circHIAT1, forward: CCCAGTCTTCCATCAACTG, reverse: CATCGGAAACCCTGGATATT; B2 M, forward: AGATGAGTATGCCTGCCGTG, reverse: GCGGCATCTTCAAACCTCCA. B2 M was used as the reference gene. The relative expression and fold change of each gene were calculated using the Pfaffel method ($E^{‐\Delta \Delta CT}$). The products of real‐time qPCR were validated in agarose with a concentration of 2% in TBE buffer by use of an electrophoresis system.

### 2.5 Statistical analysis

All statistical analyses were performed by the use of SPSS 24.0 software (SPSS Inc., Chicago, IL, USA) and GraphPad Prism 8.4.3 software (GraphPad Software, La Jolla, CA, USA). The normal distributed continuous variable was presented as mean value ±standard deviation. To check the normal distribution of samples, Shapiro‐Wilk test was applied. The expression of candidate circRNAs in this study had a normal distribution. Comparison of relative expression of circRNAs between tumor tissue and BPH tissue was calculated by independent‐sample t test, and in relation to the effect size measured through Cohen's D index. Mann‐Whitney test was used for independent variables with two classes or two states. In this regard, the relationship of circCDR1AS and circHIAT1 (Δ∁τ) expression levels with gender, DRE (normal‐abnormal), PSAD (≤ 0.15‐> 0.15), and bone metastasis (negative‐positive) was determined through Whitney test, along with the corresponding effect size measured by correlation coefficient *r* ($r=\frac{z}{\sqrt{n}}$), while we applied Kruskal‐Wallis test for calculating the relationship between studied circRNAs and BMI, total PSA, free/total PSA ratio, GS, and ISUP. Finally, the receiver‐operating characteristic (ROC) curve and the area under the ROC curve (AUC) were used to evaluate the clinical diagnostic value of candidate circRNAs separately (ie, ROC curve for separate circRNAs and PSA values) and ROC curves in logistic regression to determine the best AUC of two circRNAs together or in combination with PSA to differentiate PCa tumor tissue from BPH (related effect size was measured by McFadden R squared). For all statistical evaluations, the *p* value <0.05 was considered as a significant measure.

## 3 RESULTS

The PCa patients and BPH subjects had a median age of 67 (range 56–87) and 71 (range 56–93), respectively. There was a significant difference between the two groups in terms of DRE status, in which 56% of patients vs 16% of BPH individuals were abnormal (*p* = 0.004). The two groups also had significant differences in amount of PSA (*p* < 0.001). Distributions of PSA levels in patients were as follows: 4.35% less than 4 ng/ml, 21.73% 4–9.9 ng/ml, 26.09% 10–20 ng/ml, and 47.83% with a PSA >20 ng/ml, while this distribution of PSA in BPH individuals were 28.57% less than <4 ng/ml, 47.62% 4–9.9 ng/ml, 14.29% 10–20 ng/ml, and 9.52% with a PSA >20 ng/ml. Regarding the PSAD, its level was higher in patients than in BPH subjects (*p* = 0.004), in which 60.87% of patients showed values above 0.15 ng/ml/cm^3^ and 39.13% ≤0.15 ng/ml/ cm^3^. Among BPH patients, 81.82% were with PSAD ≤0.15 ng/ml/cm^3^ and 18.18% with a PSAD >0.15 ng/ml/cm^3^. In addition, when the patients were grouped according to the Gleason scores, 16% of patients had a value of ≤6, 48% with a score of 7, and the remaining 36% indicated a value ≥8. Other baseline characteristics were listed in Table 1. It should be noted that the data of some clinicopathological variables, including PSA levels, free/total PSA ratio, PSA density, and bone scan, were not available in the medical records of participants, and therefore, their information displayed in the Table 1 is not complete.

### 3.1 **Expression levels of circCDR1AS and circHIAT1 in prostate samples of subjects and their correlation**s **with clinical characteristics**

**FIGURE 1:**
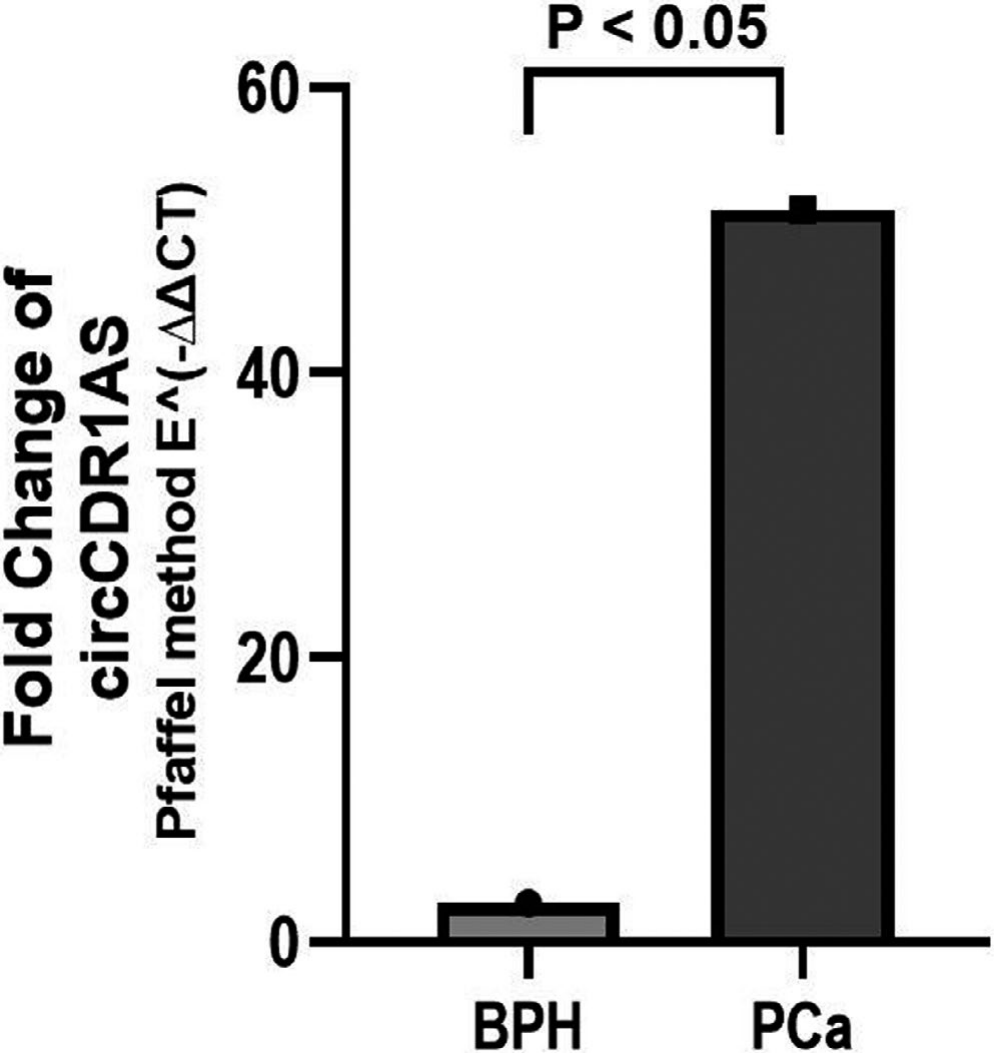
Relative expression of circCDR1AS in prostate tumor tissue vs BPH. The comparison of expression levels of circCDR1AS in PCa tissue (*n* = 25) vs BPH tissue (*n* = 25) was done using parametric independent‐sample t test. The expression level of circCDR1AS was significantly upregulated in 18.74% PCa tissues compared with the BPH tissues. The mean of fold change of each group was calculated using the Pfaffel method ($E^{‐\Delta \Delta CT}$). The *p* < 0.05 was considered as a significant level. PCa, prostate cancer. BPH, benign prostatic hyperplasia

**FIGURE 2:**
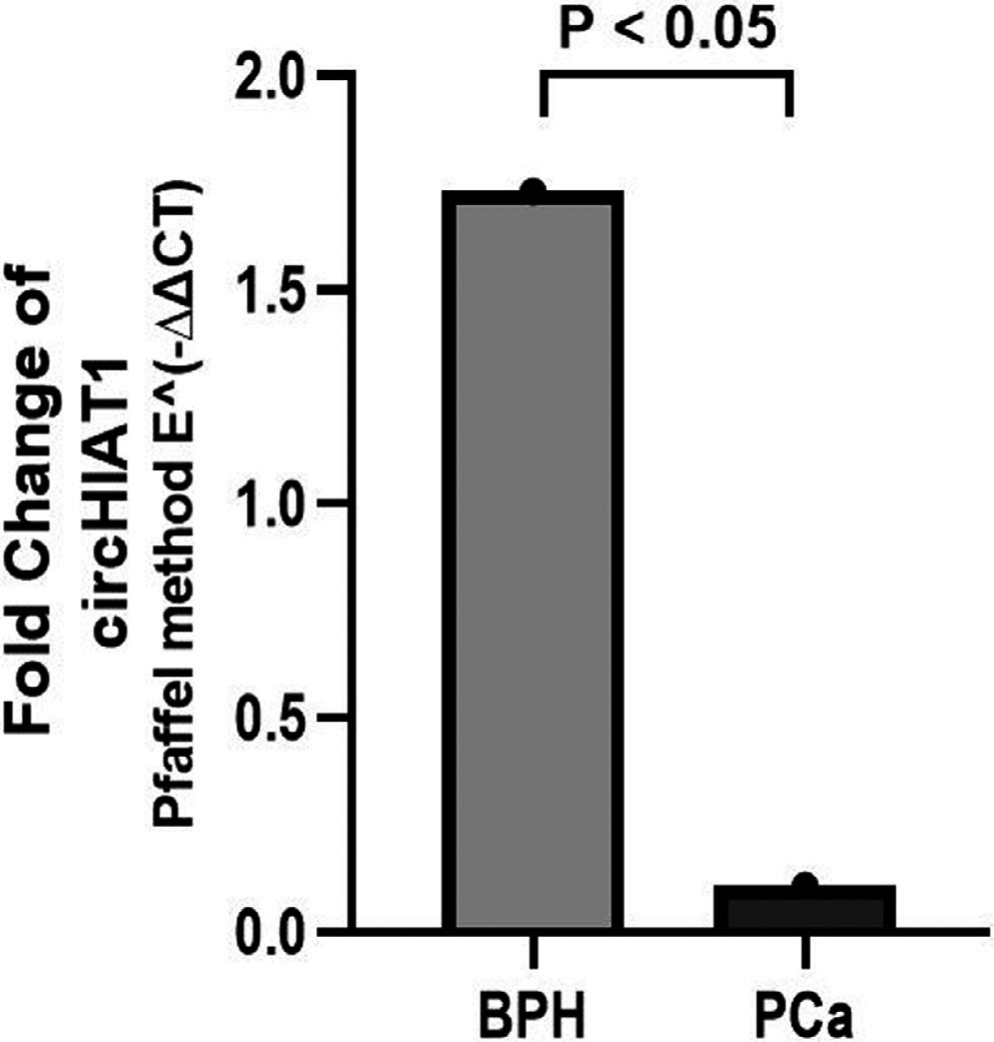
circHIAT1 relative expression in prostate tumor tissue vs BPH. The comparison expression levels of circHIAT1 in PCa tissue (*n* = 25) vs BPH tissue (*n* = 25) was performed using parametric independent‐sample t test. The expression level of circHIAT1 was significantly downregulated in 0.06% PCa tissues compared with the BPH tissues. The mean of fold change of each group was determined using the Pfaffel method ($E^{‐\Delta \Delta CT}$). The *p* < 0.05 was considered as a significant measure. PCa, prostate cancer. BPH, benign prostatic hyperplasia

Our evaluations indicated that expression of circCDR1AS was substantially increased in PCa tissues compared with the BPH tissues (Cohen's D = 1.395, Power ≥99%, *p* < 0.05, Figure 1). Additionally, it was found that the expression level of the circHIAT1 in PCa tumor tissue is considerably lower than BPH tissues (Cohen's D = 1.316, Power ≥99%, *p* < 0.05, Figure 2). In subsequent analyzes, we evaluated the relationship between the expression level of these two circRNAs and different study variables. In this regard, we observed that in the patient group and not in the group of BPH, individuals over the age of 60 have an increased expression of the circCDR1AS expression compared with individuals with the age ≤60 (*r* = −0.475, *p* < 0.05). Other variables showed no relationship with expression levels of this circRNA (Table 2). Also, as shown in Table 3, no relationship was found between the study variables and the change in circHIAT1 expression.

**TABLE 2:** Association between circCDR1AS expression level (Δ∁τ) and clinical parameter in PCa patients and BPH controls

Characteristics	Subgroup	CircCDR1AS Number (Mean ±SD (std. error))			
		BPH	*p* value	PCa	*p* value
Age (years)	≤ 60	4 (−3.21 ± 3.51 (1.76))	0.415	5 (−7.24 ± 1 (0.45))	**0.017**
	> 60	21 (−1.00 ± 4.07 (0.89))		20 (−5.34 ± 1.87 (0.42))	
BMI (kg/m²)	18.5–24.9	6 (1.32 ± 5.7 (2.33))	0.266	11(−5.09 ± 2.3 (0.69))	0.203
	25–29.9	11 (−1.74 ± 2.86 (0.86))		9 (−6 ± 10.45 (0.48))	
	≥ 30	8 (−2.86 ± 3.32 (1.17))		5 (−6.62 ± 1.25 (0.56))	
DRE	Normal	21 (−1.62 ± 4.17 (0.91))	0.459	11 (−6.01 ± 1.76 (0.53))	0.702
	Abnormal	4 (−0.02 ± 3.06 (1.53))		14 (−5.5 ± 2 (0.54))	
Total PSA (ng/ml)	<4	6 (−2.66 ± 4.47 (1.82))	0.770	1 (−7.92)	0.387
	4–9.9	10 (−0.45 ± 4.88 (1.54))		5 (−5.35 ± 1.36 (0.61))	
	10–20	3 (−0.47 ± 2 (1.16))		6 (−6.28 ± 1.63 (0.66))	
	> 20	2 (−1.36 ± 0.05 (0.03))		11 (−5.84 ± 1.97 (0.6))	
Free/total PSA ratio (ng/ml %)	≤ 10	3 (0.44 ± 3.58 (2.06))	0.406	6 (−5.72 ± 1.54 (0.63))	0.303
	11–18	2 (−2.08 ± 3.82 (2.7))		7 (−2.9 ± 2.12 (0.80))	
	18.1–25	2 (−3.66 ± 3.3 (2.34))		1 (−4.51)	
	> 25	3 (−3.54 ± 1.65 (0.95))		0	
PSA density (ng/ml/cm^3^)	≤ 0.15	18 (−1.63 ± 4.4 (1.04))	0.798	9 (−5.08 ± 1.68 (0.56))	0.413
	> 0.15	4 (−1.24 ± 2.25 (1.12))		14 (−6.10 ± 2.01 (0.54))	
Gleason score (n/%)	≤ 6	‐		4 (−6.59 ± 1.40 (0.7))	0.130
	7	‐		12 (−6.01 ± 2.20 (0.63))	
	≥ 8	‐		9 (−4.95 ± 1.44 (0.48))	
ISUP (n/%)	1	‐	‐	4 (−6.59 ± 1.40 (0.70))	0.225
	2	‐		1 (−6.80)	
	3	‐		11 (−5.95 ± 2.29 (0.70))	
	4	‐		9 (−4.95 ± 1.43 (0.48))	
Bone metastasis	Negative	‐	‐	14 (−6.27 ± 1.78 (0.48))	0.052
	Positive	‐		5 (−3.84 ± 1.75 (0.78))	

Note

Data were presented as mean value ±standard deviation or count. *p* < 0.05 was considered significant (in bold).

**TABLE 3:** Association between circHIAT1 expression level (Δ∁τ) and clinical parameter in PCa patients and BPH controls

Characteristics	Subgroup	CircHIAT1 Number (Mean ± SD (Std. Error))			
		BPH	*p* value	PCa	*p* value
Age (years)	≤60	4 (−1.52 ± 5.55 (2.77))	0.941	5 (4.47 ± 2.06 (0.92))	0.089
	>60	21 (−0.72 ± 3.35 (0.73))		20 (2.87 ± 2.46 (0.55))	
BMI (kg/m²)	18.5–24.9	6 (2.59 ± 2.25 (0.92))	0.15	11 (3.44 ± 2.76 (0.83))	0.715
	25–29.9	11 (−2.87 ± 3.67 (1.11))		9 (2.61 ± 2.44 (0.81))	
	≥30	8 (−0.66 ± 2.50 (0.88))		5 (3.95 ± 01.97 (0.88))	
DRE	Normal	21 (−0.38 ± 3.41 (0.74))	0.208	11 (2.61 ± 2.27 (0.68))	0.324
	Abnormal	4 (−3.3 ± 4.43 (2.21))		14 (3.75 ± 2.57 (0.69))	
Total PSA (ng/ml)	<4	6 (0.03 ± 2.99 (1.22))	0.548	1 (6.84)	0.158
	4–9.9	10 (−0.48 ± 3.36 (1.06))		5 (2.85 ± 2.3 (1.03))	
	10–20	3 (−3.4 ± 5.11 (2.95))		6 (3.94 ± 1.34 (0.0.55))	
	>20	2 (−2.45 ± 1.80 (1.27))		11 (2.38 ± 2.88 (0.86))	
Free/total PSA ratio (ng/ml %)	≤10	3 (−3.17 ± ± 5.14 (3.12))	0.643	6 (3.26 ± 1.88 (0.77))	0.121
	11–18	2 (0.55 ± 3.52 (2.49))		7 (0.52 ± 2.4 (0.91))	
	18.1–25	2 (−2.88 ± 2.41 (1.7))		1 (1.44)	
	>25	3 (−0.74 ± 2.45 (1.4))		0	
PSA density (ng/ml/cm^3^)	≤0.15	18 (−0.55 ± 3.65 (0.86))	0.551	9 (3.21 ± 2.22 (0.74))	0.801
	>0.15	4 (−2.25 ± 4.77 (2.38))		14 (3.10 ± 2.74 (0.73))	
Gleason score (n/%)	≤6	‐	‐	4 (4.83 ± 1.95 (0.97))	0.245
	7	‐		12 (2.99 ± 2.35 (0.68))	
	≥8	‐		9 (2.89 ± 2.77 (0.92))	
ISUP (n/%)	1	‐	‐	4 (4.83 ± 1.95 (0.97))	0.140
	2	‐		1 (−1.08)	
	3	‐		11 (3.36 ± 2.07 (0.62))	
	4	‐		9 (2.89 ± 2.77 (0.92))	
Bone metastasis	Negative	‐	‐	14 (3.22 ± 2.2 (0.59))	0.643
	Positive	‐		5 (4.30 ± 3.26 (1.46))	

Note

Data were presented as mean value ±standard deviation or count. *p* < 0.05 was considered significant (in bold).

### 3.2 Potential diagnostic values of circCDR1AS and circHIAT1 for PCa

**FIGURE 3:**
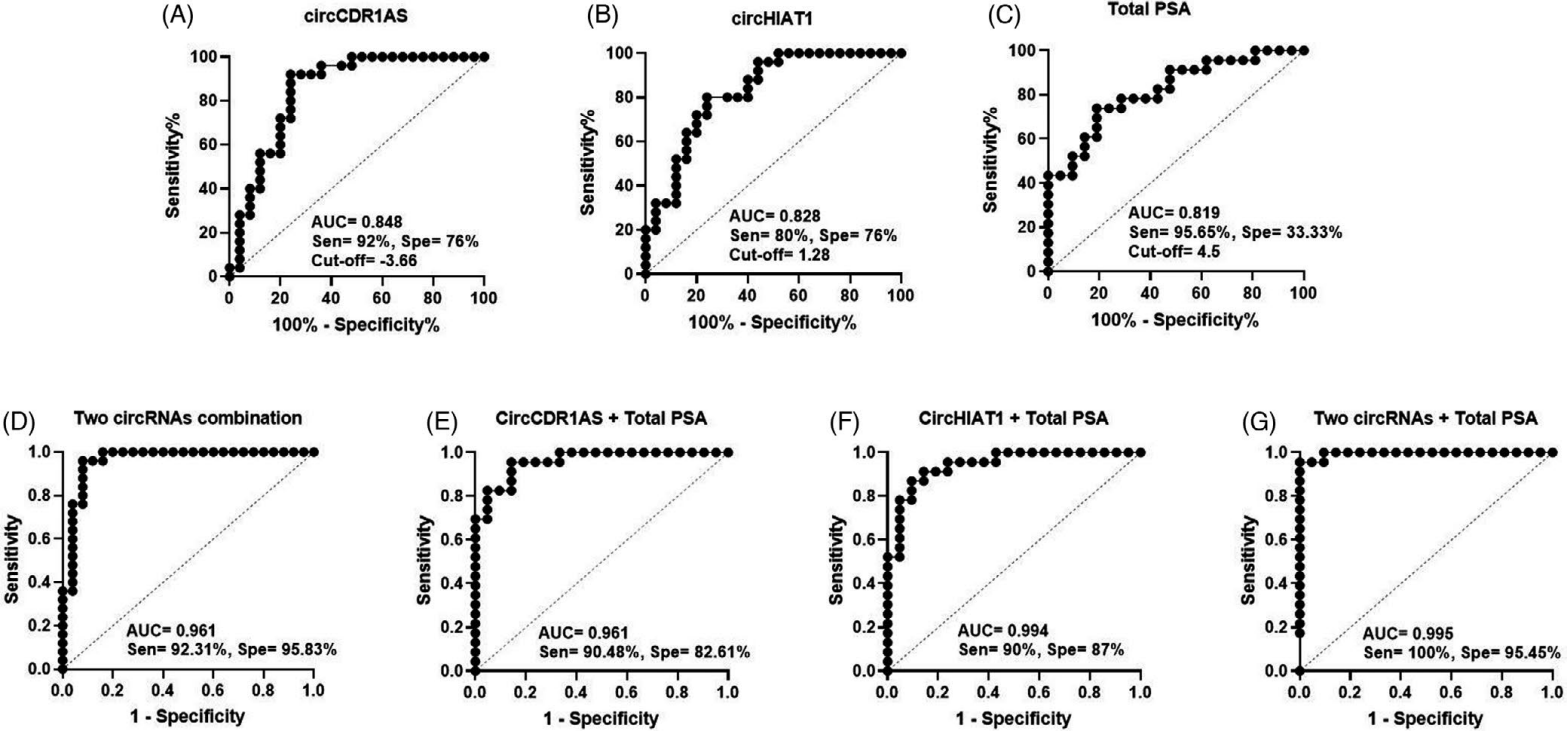
ROC curve analysis for evaluating the biomarker value of circCDR1AS, circHIAT1, and total PSA. The diagram shows the biomarker potential of candidate circRNAs expression in PCa patients and BPH controls. (A, B, C) The ROC curve displays the relatively good value of circCDR1AS, circHIAT1, and total PSA in distinguishing of PCa from BPH (${p<0.0001}_{A.B}$, ${p<0.0003}_{C}$). (D, E, F) The ROC curve analysis of combined model for circCDR1AS and total PSA as well as circHIAT1 and total PSA (${p<0.0001}_{D.E.F}$). (G) ROC curve of combined model of both candidate circular RNAs and total PSA shows the biomarker value for distinguishing PCa from BPH (*p* < 0.001). The ROC curve, receiver‐operating characteristic curve

We further assessed the diagnostic effectiveness of candidate circRNAs to differentiate PCa tumor tissue from BPH through ROC curve. The observations indicated that the AUC of circCDR1AS was 0.848, with a cutoff value of −3.66 (Δ∁τ) as well as the 92% sensitivity and 76% specificity ((95% CI (0.73–0.96), Std. Error 0.57, *p* < 0.0001, Figure 3A). The ROC data of the circHIAT1 showed an AUC of 0.828, cutoff value of 1.28 (Δ∁τ) and the 80% sensitivity and 76% specificity (95% CI (0.41–0.94), Std. Error 0.57, *p* < 0.0001, Figure 3B). Regarding the total PSA, we found an AUC of 0.819 (with a cutoff value of 4.5) and the sensitivity and specificity of 95.65% and 33.33%, respectively (95% CI (0.70–0.94), Std. Error 0.62, *p* < 0.0003, Figure 3C). However, when the combinations of the two circRNAs were analyzed together, a higher AUC (0.961) was obtained than total PSA alone ((95% CI (0.91–1), Std. Error 0.28, $R_{\mathrm{McF}}^{2}$ = 0.6825, *p* < 0.0001, Figure 3D). Furthermore, when the circCDR1as combined with total PSA, the sensitivity and specificity were 90.48% and 82.61%, respectively (AUC of 0.961, 95% CI (0.73–0.096), Std. Error 0.57, $R_{\mathrm{McF}}^{2}$ = 0.6258, *p* < 0.0001, Figure 3E). Correspondingly, when we combined the circHIAT1 with total PSA, the sensitivity and specificity were 90% and 87%, respectively (AUC of 0.994, 95% CI (0.73–0.096), Std. Error 0.57, $R_{\mathrm{McF}}^{2}$ = 0.5706, *p* < 0.0001, Figure 3F). Therefore, in the combined model of one of the two selected circRNAs with total PSA, the sensitivity and AUC were better than total PSA alone, but not specificity. In line with these observations, the results showed that the AUC of three combined factors (circCDR1AS, circHIAT1, and total PSA) was 0.995 with the sensitivity and specificity of 100% and 95.45%, respectively (95% CI (0.98–1), Std. Error 0.005, $R_{\mathrm{McF}}^{2}$ = 0.9046, *p* < 0.0001, Figure 3G). Therefore, by combining two candidate circRNAs with total PSA, the highest biomarker values can be obtained to differentiate PCa from BPH.

## 4 DISCUSSION

Heterogeneous phenotypes of prostate tumors are a reflection of their heterogeneous genetic changes, which also have an important effect on its physiopathology, treatment, and diagnosis.^26^, ^27^, ^28^, ^29^ Thus, revealing PCa‐related genetic signatures could be useful in identifying the molecular etiology of the tumors as well as introducing new diagnostic, therapeutic, and prognostic biomarkers.^26^, ^27^, ^28^, ^30^ In this study, we investigated the expression levels of two key circRNAs including the circCDR1AS (hsa_circ_0001946) and circHIAT1 (hsa_circ_0000096) in prostate tumor tissues compared with BPH samples as well as their correlations with clinicopathological characteristics of the tumors.

Our results showed that the circCDR1AS had a significantly increased expression level in prostate tumors compared with BPH tissues. Notably, evidence from previous studies suggests an oncogenic function for circCDR1AS through regulating some key oncogenic signaling pathways in tumor hallmarks.^21^, ^31^, ^32^ Although data on the exact function of circCDR1AS are not yet fully understood, some clues suggest a role for this circRNA through sponging the specific miRNAs in a complex ceRNA regulatory networks for regulating some target mRNAs at the post‐transcriptional level, and therefore, its expression abnormalities in these networks lead to tumorigenesis.^17^, ^33^ For example, a study found that the expression level of this circRNA in metastatic PCa cell lines (PC3) is 200 times higher than in RWPE‐1 normal prostate epithelial cells lines and 22RV1 prostate carcinoma epithelial cells, and its bioinformatic data showed that circCDR1AS could potentially play its regulatory role through a circRNA‐miRNA interaction network by acting on the mir‐7‐5p.^34^ A new study conducted by Niu Y et al on the prostate cancer cell lines (LNCaP, 22Rv1, and PC‐3) revealed that this circRNA executes its abnormal roles in the invasion and migration of the tumor cells via circCDR1as/miR‑641/XIAP regulatory axis, and during tumor phenotypes, the expressions of circCDR1AS and XIAP were upregulated, while the expression of miR‐641 was reduced in these cell lines compared with the normal prostate epithelial cell line (RWPE‐1).^17^ In colorectal cancer (CRC), overexpression of circCDR1AS has been shown to be associated with tumor size, lymph node metastasis, and poor overall survival (OS) in patients.^35^ Haiyan et al found an elevated expression of this circRNA in gastric cancer in correlation with more advanced tumor stages, distant metastasis, and poor survival in patients.^15^

Another report showed that circCDR1AS is overexpressed as an oncogene in HCC and executes its role through suppressing miR‐7 in a PTEN/PI3K/AKT‐related signaling network and functionally stimulates proliferation and invasion of HCC cells.^36^ Moreover, its expression was clinically associated with a more malignant form of HCC patients.^36^ Therefore, our findings and such evidence suggests that the circCDR1AS plays a key role in the development and etiology of these cancers PCa, as well as their pathophysiology by activating oncogenic signaling pathways including PTEN/PI3K/AKT via inhibiting the mir‐7. Another finding of the present study was limited to case group in which the PCa patients over 60 years of age had a higher expression of the circCDR1AS than patients ≤60 years of age. Age is a known risk factor for PCa, so as the age increases, the risk of developing this cancer increases.^37^ Thus, this expression signature may be age‐dependent event, and it can be thought that one of the possible molecular mechanisms behind the relationship of increasing age with increasing risk of PCa is the elevating of circCDR1AS, although this observation needs to be studied in more detail.

We furthermore found that the expression level of circHIAT1 in the tumors was reduced compared with BPH tissues. This result is important because previous data showed that this circRNA has a tumor‐suppressive function and its reduced expression plays a prominent role in the development of some cancers.^14^, ^38^ Li et al. reported that circHIAT1 is considerably downregulated in gastric tumor tissues and gastric cancer cell lines,^38^ and mechanistically, circHIAT1 involved in controlling of proliferation and migration ability of gastric tumor cells by inhibiting the expression of cell cycle and migration‐related protein‐encoding genes at both in vitro and in vivo by regulating the miR‐224 and miR‐200a.^38^ In another work on ccRCC, it was found that the expression of circHIAT1 is downregulated by AR and through suppressing its host gene named HIAT1 at the transcriptional level via a regulatory axis involving of the circ_HIAT1 and miR‐195‐5p/29a‐3p/29c‐3p/CDC42.^14^ Moreover, this circHIAT1 downregulation was in relation to some important clinical features including, migration and metastasis of tumors as well as a worse overall survival of ccRCC patients.^14^ The AR has been found to play important roles in progression and metastasis of the PCa through the targeting of miR‐195‐5p/29a‐3p/29c‐3p/CDC42 signaling pathway via AR/circHIAT1.^14^ Therefore, according to the findings of our study and also these reported data about the role of circHIAT1 in the pathology of other tumors, it seems that this circRNA also plays a key role in the development and pathophysiology of PCa.

Finally, we determined the potential diagnostic biomarker values of both circCDR1AS and circHIAT1 for PCa, especially with the aim of differentiating BPH from PCa. Regarding the circCDR1AS, ROC curve analysis showed an AUC of 0.848 with 92% sensitivity and 76% specificity. Also, it was found an AUC of 0.828 with 80% sensitivity and 76% specificity for circHIAT1. However, these data for the total PSA were an AUC of 0.819 as well as the sensitivity and specificity of 96% and 33%, respectively. Notably, when the biomarker value data were combined as the circHIAT1+circCDR1AS and also in the form of three factors including circCDR1AS+circHIAT1+total PSA, the higher values of AUC, sensitivity, and specificity were obtained than the total PSA alone. Therefore, these two circRNAs are expected to be potentially important biomarkers for PCa screening and diagnosis, although, this hypothesis needs further research.

In summary, the findings of current study indicated that in patients with PCa, compared with BPH individuals, the expression levels of circCDR1AS increased but the circHIAT1 decreased. Also, the expression of circCDR1AS in the PCa group showed a significant relationship with the increasing age, and finally, we indicated the potential biomarker potential of the molecular signature of these two circRNAs to differentiate PCa from BPH. Such data suggest that aberrant expressions of circCDR1AS and circHIAT1 are the two different molecular pathologies that occur in PCa and on the contrary show potential biomarker value, although further studies are needed. Of course, it should be noted that this study also had some limitations, including the small sample size of the study population, as well as the impossibility of using adjacent prostate tumor tissue samples and performing functional analyzes of target circRNAs. Therefore, by removing these limitations and conducting additional studies, the exact roles of these two circRNAs in the pathophysiology of PCa can be revealed, which itself develops their clinical practical values in the future.

## CONFLICT OF INTEREST

The authors declare that they have no conflict of interest.

## AUTHOR CONTRIBUTIONS

Mahsa Aghajani Mir carried out the development and management of the project, sample collection, and data analysis, and wrote the article. Emadoddin Moudi collected the samples. Nima Motamed Management data analysis. Abdolreza Daraei and Hossein Dinmohammadi were responsible for project management and article editing. The authors have read and approved the final article.

## Data Availability

All data are included in this article.
